# Acute myocardial infarction induces remodeling of the murine superior cervical ganglia and the carotid body

**DOI:** 10.3389/fcvm.2022.758265

**Published:** 2022-10-06

**Authors:** Yang Ge, Lieke van Roon, Janine M. van Gils, Tom Geestman, Conny J. van Munsteren, Anke M. Smits, Marie José T. H. Goumans, Marco C. DeRuiter, Monique R. M. Jongbloed

**Affiliations:** ^1^Department of Anatomy and Embryology, Leiden University Medical Center, Leiden, Netherlands; ^2^Department of Cardiology, Leiden University Medical Center, Leiden, Netherlands; ^3^Department of Nephrology, Leiden University Medical Center, Leiden, Netherlands; ^4^Department of Cell and Chemical Biology, Leiden University Medical Center, Leiden, Netherlands

**Keywords:** myocardial infarction, superior cervical ganglion, carotid body, neurotrophic factors, GAP43, neuronal remodeling, BDNF (brain derived neurotrophic factor), NGF (nerve growth factor)

## Abstract

A role for cardiac sympathetic hyperinnervation in arrhythmogenesis after myocardial infarction (MI) has increasingly been recognized. In humans and mice, the heart receives cervical as well as thoracic sympathetic contributions. In mice, superior cervical ganglia (SCG) have been shown to contribute significantly to myocardial sympathetic innervation of the left ventricular anterior wall. Of interest, the SCG is situated adjacent to the carotid body (CB), a small organ involved in oxygen and metabolic sensing. We investigated the remodeling of murine SCG and CB over time after MI. Murine SCG were isolated from control mice, as well as 24 h, 3 days, 7 days and 6 weeks after MI. SCG and CBs were stained for the autonomic nervous system markers β3-tubulin, tyrosine hydroxylase (TH) and choline acetyltransferase (ChAT), as well as for the neurotrophic factors brain derived neurotropic factor (BDNF), nerve growth factor (NGF) and their tyrosine receptor kinase (pan TRK). Results show that after MI a significant increase in neuron size occurs, especially in the region bordering the CB. Co-expression of TH and ChAT is observed in SCG neuronal cells, but not in the CB. After MI, a significant decrease in ChAT intensity occurs, which negatively correlated with the increased cell size. In addition, an increase of BDNF and NGF at protein and mRNA levels was observed in both the CB and SCG. This upregulation of neurotropic factors coincides with the upregulation of their receptor within the SCG. These findings were concomitant with an increase in GAP43 expression in the SCG, which is known to contribute to axonal outgrowth and elongation. In conclusion, neuronal remodeling toward an increased adrenergic phenotype occurs in the SCG, which is possibly mediated by the CB and might contribute to pathological hyperinnervation after MI.

## Introduction

About one third of all global deaths are attributed to cardiovascular diseases (1). In western countries, the incidence of sudden cardiac death (SCD) is 50–100 per 100,000 which is attributed to coronary artery disease (CAD) in 70–80% of cases, despite the development of reperfusion strategies and medical therapies (2). SCD after myocardial infarction (MI) has been classically linked to heterogeneous conduction in the infarct border zone caused by surviving cardiomyocytes surrounding the scar area, resulting in polymorphic ventricular tachycardia (VT) based on micro-re-entry (3, 4). Interestingly, in the past decades a role for the cardiac autonomic nervous system in arrhythmogenesis after MI has increasingly been recognized (5, 6). The heart is innervated by the autonomic nervous system, divided in sympathetic and parasympathetic branches, regulating cardiac function. In order to maintain a regular heartbeat, a balance is needed between sympathetic and parasympathetic tone. Parasympathetic input toward the heart is provided by (branches of) the vagal nerve that synapse in parasympathetic ganglia at the epicardial layer of the heart. Preganglionic cardiac sympathetic axons synapse with postganglionic sympathetic neurons in the sympathetic chain (7). In humans, cardiac input from the sympathetic chain is provided by both cervical as well as thoracic contributions (7, 8).

A myriad of studies have reported a potential association of cardiac sympathetic hyperinnervation, usually defined as an increased density of sympathetic nerve fibers in the area of damage, with SCD after MI. To date the exact underlying mechanism of the relation between sympathetic hyperinnervation and VT after MI is still uncertain. Likely, factors secreted by the ischemic myocardium retrogradely stimulate axonal outgrowth and remodeling of sympathetic ganglia, altering electrophysiological properties, thereby increasing the risk of VT and SCD (9–11). Recent data shows an upregulation of nerve growth factor (NGF) in the ischemic zone after MI, that supports this concept (12).

Although several studies have shown sympathetic hyperinnervation as well as neuronal remodeling after MI, the exact timeline of this phenomenon is less clear. In several species, neuronal remodeling has been described to occur 1–8 weeks after MI, characterized by increased expression of growth associated protein (GAP43) and synaptophysin – both markers for neuronal outgrowth – and increased amounts of tyrosine hydroxylase (TH), suggesting an increase in innervation and a switch toward a more adrenergic phenotype (13–16). Most studies, however, focus on the stellate ganglion, whereas limited information is available on the relevance of the other ganglia providing sympathetic input to the heart.

The superior cervical ganglion (SCG) gives input to the carotid plexus whose fibers run along the carotid arteries and provide sympathetic input toward the head where it stimulates parts of the eye, mouth and small blood vessels. The SCG also participates in innervation of the heart, providing the superior cardiac nerve that joins with postganglionic sympathetic fibers originating from other sympathetic ganglia at the cardiac plexus (7, 8). Remarkably, in mice it has been shown that ganglionectomy of the SCG before MI leads to an almost entire loss of myocardial sympathetic innervation of the left ventricular anterior wall, in addition to a significantly reduction in chronic consequences of MI, such as myocardial inflammation, myocyte hypertrophy, and overall cardiac dysfunction (17). In human, it has been established that the SCG is involved in cardiac innervation (8), although the impact of a potential remodeling of this ganglion after MI, is unclear. In this respect, it may be relevant that the SCG is situated adjacent to the carotid body (CB), a small organ involved in oxygen, carbon and pH sensing, that has been shown to produce many neurotrophic factors (18). Of interest, Rocha et al. report that in rabbits the response of the chemo sensitive cardiac reflex of the CB was enhanced in the acute phase of MI (19). Hypertonicity of the CB has been linked with cardiac disease such as hypertension and chronic heart failure. In rats with induced chronic heart failure, denervation of the CB performed early after MI, resulted in improved survival due to reduction of ventricular remodeling, less fibrosis and reduction of arrhythmias (20). Whether this is a transient phenomenon is unclear, as is the time-course of remodeling of the superior cervical ganglion and CB after MI.

Given the relevance of the SCG in cardiac innervation of the murine heart, as well as the still enigmatic role of this ganglion and the CB in the innervation of the human heart in health and disease, in the current study we investigated the remodeling of the murine SCG as well as the bordering CB over time after MI.

## Materials and methods

### Animals

C57BL/6J (Jackson Laboratory) male mice of 13 weeks old (*n* = 20) were used. All animal experiments were approved by the Animal Ethics Committee of the Leiden University (License number AVD1160020185325), Leiden, The Netherlands. All mice were maintained in a specific pathogen-free facility on a 12 h day and night cycle and regularly monitored.

### Induction of myocardial infarction and superior cervical ganglia isolation

**FIGURE 1:**
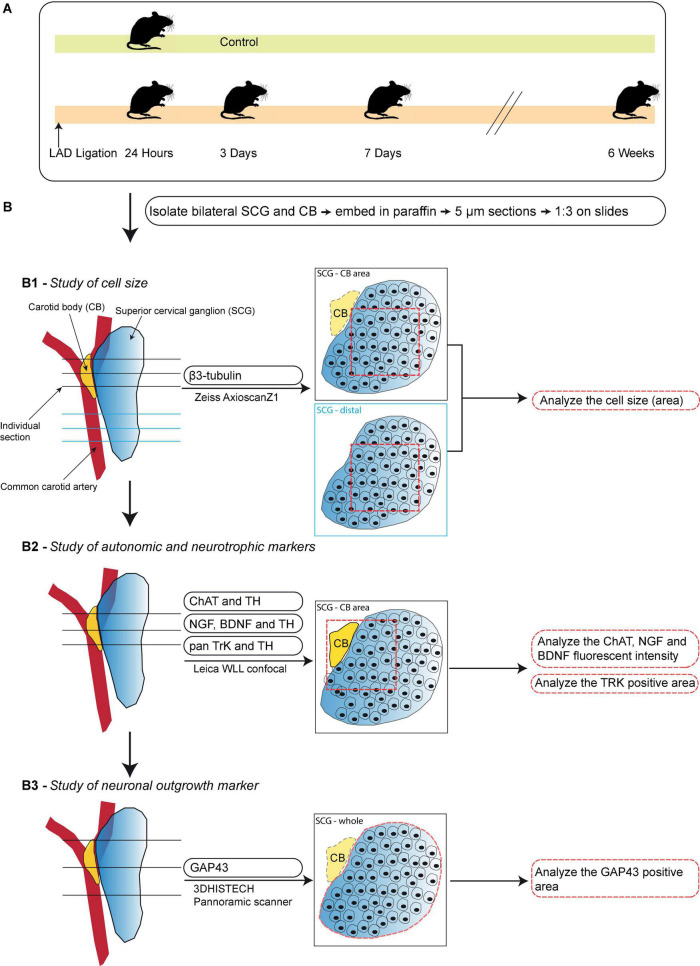
Schematic workflow of the time-course study. **(A)** Schematic overview of the studied timepoints after induction of MI. **(B)** A step-by-step illustration of the performed immunofluorescence stainings in this timepoint study of the SCG and CB after MI. Left panels display an overview of the selection of sections for each analysis. The midline panels explain the regions within the sections that were selected for each quantification method. **(B1)** Analysis of neuronal size in the SCG-CB area and SCG-distal area. **(B2)** Analysis of the fluorescent intensity of autonomic and neurotrophic markers in the SCG-CB area. **(B3)** Analysis of the area of growth associated protein 43 (GAP43) in the SCG.

Myocardial infarction was induced as previously described (12). Briefly, mice were anesthetized with 2% isoflurane, intubated and ventilated. The left anterior descending coronary artery (LAD) was permanently ligated and ischemia was confirmed by discoloration of the anterior wall of the left ventricle. The mice were given the analgesic drug Temgesic, 24 h before and after the operation to relieve pain. As control, untreated mice (*n* = 4, 8 SCGs) were included. All mice were euthanized by CO_2_ asphyxiation; untreated control mice (*n* = 4; 8 SCGs) and 24 h (*n* = 5; 10 SCGs), 3 days (*n* = 3; 6 SCGs), 7 days (*n* = 5; 10 SCGs), or 6 weeks (*n* = 3; 6 SCGs) after MI (Figure 1A). The SCG and hearts were removed as previously described (21). Briefly, to dissect the left- and right SCG, an incision was made in the skin of the neck area, the submandibular glands were moved aside, whereafter the carotid artery bifurcation with the SCG could be captured bilaterally. After excision, both SCG were fixed with 4% paraformaldehyde (104005; Merck Millipore), and embedded in paraffin. The ganglion sections, 5 μm thick, were adhered in a series of 1:3 on silane adhesive slides (Klinipath KLINKP-SIL-3057) to allow different stainings to be assessed in the same ganglion.

### Immunofluorescence detection and quantification of neuronal- and neurotrophic markers

The illustration in Figure 1B shows the schematic overview of; (i) studied markers; (ii) used microscope and scanners; (iii) location of the sections; (iv) quantification method; and (v) output parameters. All slides were deparaffinized prior to the antigen retrieval, by heating slides in Tris-EDTA buffer (pH 9, 98°C) for 12 min, and incubated with primary antibodies overnight at 4°C.

To study the cell size and autonomic nerve markers (Figures 1B1,B2), every third slide was incubated with primary antibodies: anti-tyrosine hydroxylase (TH, a marker for sympathetic nerves) (Fisher Scientific PA14679; 1:1,000), anti-choline acetyltransferase (ChAT, considered a marker for parasympathetic nerves) (Abcam ab181023; 1:1,000) and anti-β-tubulin III (β3-tubulin, a general nerve marker) (Santa Cruz; SC-80005; 1:1,000). To study neurotropic markers and receptors (Figure 1B2), one slide per ganglion with paraffin sections that contained the CB were used. The slide was incubated with the primary antibodies: anti-brain derived neurotrophic factor (BDNF) (Abcam ab108319; 1:250), anti-nerve growth factor (NGF) (Abcam ab6199; 1:100) or anti-pan tyrosine receptor kinase (pan TrK) (Abcam ab181560; 1: 500) combined with anti-TH (Fisher Scientific PA14679; 1:1,000).

On the second day, the slides were incubated with their corresponding secondary antibodies: donkey anti-rabbit Alexa Fluor 488 (Invitrogen A-21206; 1:250), donkey anti-sheep Alexa Fluor 568 (Invitrogen A21099; 1:250) or donkey anti-mouse Alexa Fluor 647 (Invitrogen A31571; 1:250) for 1 h followed by a 10 min nuclear staining with DAPI (Invitrogen D3571: 1:1000). The slides were mounted with ProLong Gold Antifade Mountant (Invitrogen P36930) and the images were captured with the Zeiss AxioscanZ1 slide scanner or Leica WLL confocal microscope under the same exposure time and gain settings.

For quantification of the neuronal size (derived from the neuronal area) three sections per region were selected (Figure 1B1) and for fluorescent intensity, 3 sections per slide were selected (Figure 1B2). The individual sections were opened with ImageJ (version 1.52p) and a 500 × 500 pixels (equals 162.5 × 162.5 μm) square was placed within each section. Within each square 20 cells were drawn in as ROI and the area or intensity were measured. The intensity was corrected by subtraction of the background intensity.

### Immunohistochemical staining and quantification of growth-associated protein 43

To study neuronal outgrowth, one slide per ganglion with 5 μm-thick paraffin sections was stained with growth associated protein 43 (GAP43), a marker for nerve sprouting (22, 23) (Figure 1B3). As previously described, the sections were first deparaffinized and antigen retrieval was performed. Hereafter, the sections were incubated with the primary anti-GAP43 antibody (Abcam ab75810, 1:2,000) overnight. The next day, the primary antibody was washed away and thereafter the slides were incubated with the secondary biotinylated anti-rabbit IgG (H + L) antibody (Vector Laboratories BA-1000, 1:200) for 1 h, followed by an incubation with ABC-AP (Vector Laboratories AK-5000) for 30 min. To visualize GAP43, the slides were incubated with alkaline phosphatase (AP) substrate (Vector Laboratories SK-5105) in the dark for 5 min. The substrate was then washed away and the slides were counterstained with haematoxylin (Klinipath VWRK4085-9002) in order to visualize the nuclei. After dehydration the sections were mounted with Entellan mounting medium (Merck 107961) and all images were captured with the 3DHISTECH Pannoramic scanner.

To quantify GAP43 expression, 3 sections of each ganglion were quantified. Individual sections were imported into ImageJ and the ganglion region was selected by hand with the ImageJ selection function and set as ROI. To calculate the GAP43 + area (fractional area,%) within the SCG the measurements were performed as follows: within the green channel the total area of the ganglion was measured by setting the threshold on the maximum and this was divided by the GAP43 + area which was measured using the default threshold in ImageJ (version 1.52p).

### Hybridization chain reaction RNA fluorescent *in situ* hybridization

Hybridization chain reaction RNA fluorescent *in situ* hybridization (HCR-RNA FISH) was carried out in control and 7 days after MI SCG sections that contained the CB. The manufacturers protocol was followed and the DNA probes, DNA HCR amplifiers, hybridization buffer and wash buffer were purchased from Molecular Instruments^1^ (24). Briefly, slides were first heated for 1 h at 60°C to improve adhesion. For RNA retrieval, the slides were deparaffinized and heated in TRIS buffer for 15 min at 95°C, followed by a 10 min Proteinase K (10 μg/ml) (Promega, V3021) digestion. A humidified chamber was used in all following incubation steps. Probe hybridization with BDNF-B1 (NM_007540.4, LOT PRM659), NGF-B2 (NM_013609.3, LOT PRM660), and GAP43-B3 (NM_008083.2, LOT PRF293) was performed with 4 pmol/ml probes for 16 h at 37°C. Prior to the hairpin amplification, 6 pmol/ml of the hairpins B1-h1 + h2 (fluorophore 546), B2-h1 + h2 (fluorophore 647), and B3-h1 + h2 (fluorophore 488) were snap-cooled by heating to 95°C for 90 s and incubated for 30 min in the dark at room temperature. Sections were incubated with the hairpin amplifiers for 90 min at room temperature. To stain the nuclei, slides were incubated for 10 min with DAPI (Invitrogen, D3571; 1:1,000) and washed in PBS. Sections were mounted with Prolong Gold Antifade (Invitrogen, P36930) and imaged with a Zeiss Airyscan LSM 900 confocal microscope under the same exposure time and gain settings. mRNA expression was quantified in 3 sections per ganglion, the ganglia and CB were separately selected as ROI in ImageJ and threshold settings to measure the area were kept the same throughout the quantification. Artifacts that were highly fluorescent were manually deleted from the ROI.

### Statistics

Data are presented as mean ± standard error of the mean (SEM). One-way ANOVA and multiple comparisons followed by a Tukey’s *post hoc* analysis were used to determine statistically significant differences among groups. An unpaired Student’s was used to statistically analyze the HCR-RNA FISH data. All quantifications were performed in a blinded fashion and results were considered significantly different when the *p*-value was <0.05. GraphPad Prism (GraphPad Software, San Diego, CA, USA; version 9) was used for statistical analysis. Pearson correlation coefficients were used to test the linear relationship between two variants. R (version 4.0.2) was used for Pearson correlation coefficients and linear regression.

## Results

### Regional differences in neuronal enlargement in the superior cervical ganglia after acute myocardial infarction

To investigate the remodeling of the murine SCG over time after MI by permanent LAD ligation, SCG were collected and analyzed after 24 h, 3 days, 7 days, and 6 weeks. Although small interindividual variations in MI infarction sizes were observed, no significant differences were detected between hearts at 1 week and 6 weeks after MI (Supplementary Figures 1A,B). The presence of hyperinnervation was verified by β3-tubulin staining of the infarction region and border zone (Supplementary Figure 1A).

In order to study potential effects of sidedness, both left and right-sided ganglia were examined. Supplementary Figure 2 shows that there was no significant difference in neuronal cell size (A and B), the area of ChAT positive nuclei/total nuclei area (%) (C and D), intensity of NGF expression (E and H), intensity of BDNF expression (I and L) and GAP43 positive area (M and N) between the left and right SCG at 24 h and 7 days after MI. Since no differences could be detected between the left and right ganglia for the parameters tested in our study, ganglia were treated as independent samples onward (Supplementary Figures 2A–N).

**FIGURE 2:**
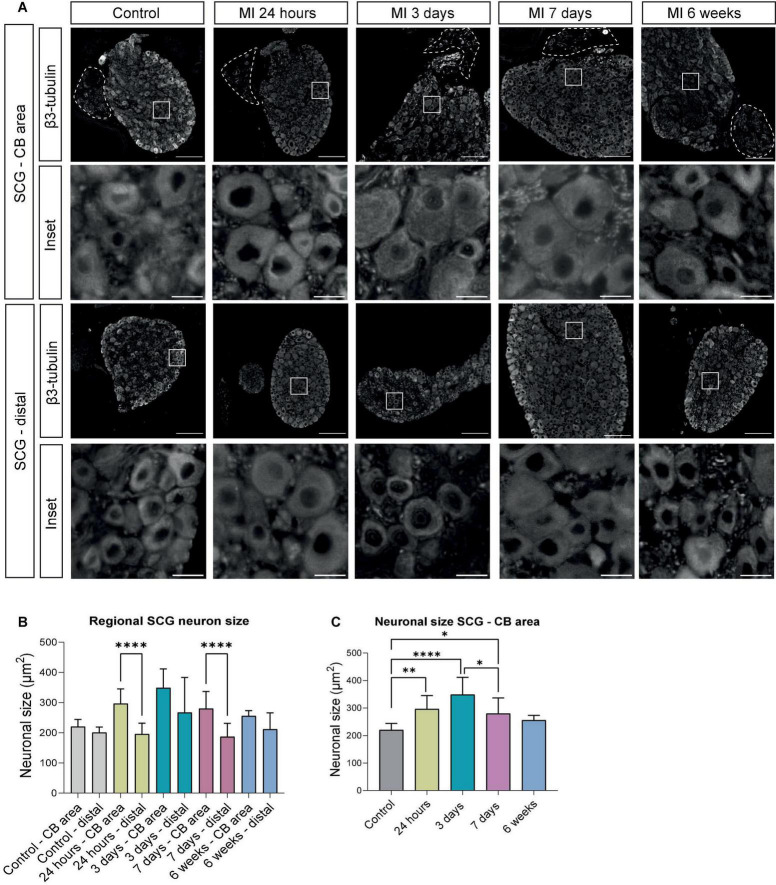
Regional differences of neuronal enlargement in the SCG after MI. **(A)** β3-tubulin immunofluorescence staining in distinct SCG regions of control mice (*n* = 4, 8 SCG) and mice 24 h (*n* = 5, 10 SCG), 3 days (*n* = 3, 6 SCG), 7 days (*n* = 6, 12 SCG), and 6 weeks (*n* = 3, 6 SCG) after MI. Scale bar indicates 100 μm in the upper panels, and 15 μm in the insets. **(B)** Bar graphs that display the alterations of the neuronal size (μm^2^) in the SCG – CB and SCG – distal region. **(C)** Bar graphs that display the alterations of neuronal size (μm^2^) in the SCG – CB area. *P < 0.05, ^**^P < 0.01, ^****^P < 0.0001.

Staining of serial sections of the murine SCG with the general nerve marker β3-tubulin provided an overview of the distribution of neurons and nerves for each timepoint (Figure 2A). Sections were studied that either contained (SCG – CB area) or lacked (SCG – distal) the CB. Quantification of the neurons in these sections revealed a significant difference in the cell size (area) at 24 h and 7 days after MI in the SCG – CB area when compared to SCG – distal area (Figure 2B). In the SCG – CB area a significant increase in cell size was observed 24 h, 3 days and 7 days after MI, when compared to control. Since we established a regional difference in neuronal enlargement, only the SCG – CB area was evaluated in this study onward.

### The area bordering the carotid body displays a decrease in choline acetyltransferase intensity

**FIGURE 3:**
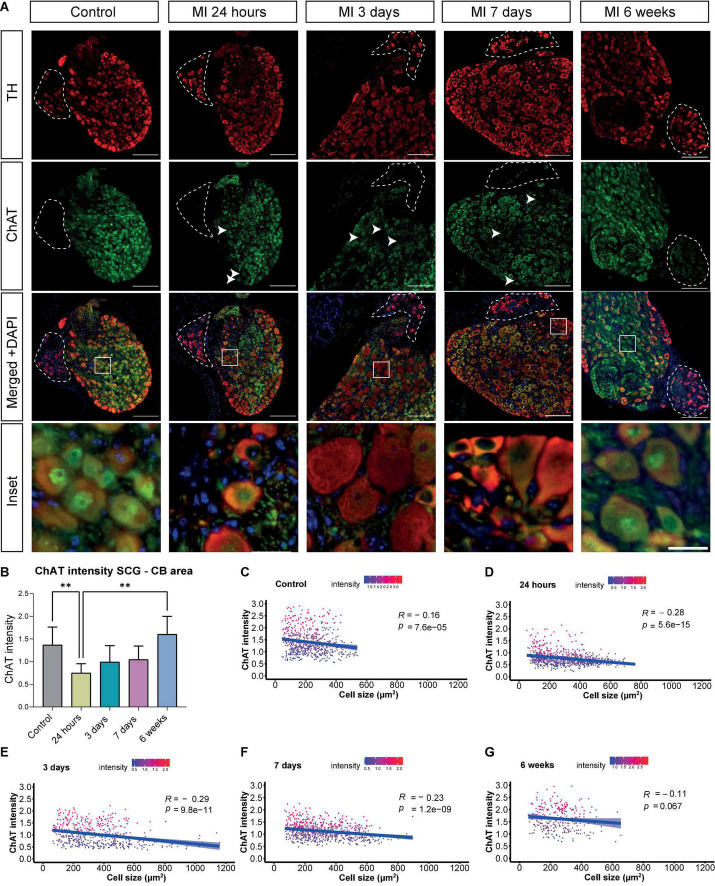
Decreased ChAT intensity in the SCG after MI. **(A)** Immunofluorescence staining of TH (red), ChAT (green) and DAPI (nuclei, blue) in the SCG-CB region of control mice (*n* = 4, 8 SCG) and mice 24 h (*n* = 5, 10 SCG), 3 days (*n* = 3, 6 SCG), 7 days (*n* = 6, 12 SCG) and 6 weeks (*n* = 2, 4 SCG) after MI. The CB is positive for TH and is indicated with dashed lines. The scalebar indicates 100 and 20 μm in the insets. **(B)** Bar graph displays the ChAT intensity in the SCG-CB region over time. **(C–G)** Linear regression analysis of the neuronal ChAT intensity plotted against the cell size of control mice and mice 24 h, 3 days, 7 days, and 6 weeks after MI. Each dot represents a single cell and the light blue regions indicate the standard error of the mean (SEM) of the ChAT intensity. The R value demonstrates the correlation coefficients at the indicated *p* values. ^**^P < 0.01.

Immunostainings of sections that contained the CB, were performed for the parasympathetic marker ChAT as well as the sympathetic marker TH at several timepoints after MI. The CB type I glomus cells could be identified as clusters of bright TH positive cells, that lack ChAT expression, bordering the SCG. The murine SCG neuronal cells co-expressed TH and ChAT in the SCG throughout the MI timeline (Figure 3A). Overall, the peripheral part of the ganglion displayed a higher TH intensity as compared to the center of the ganglion. While the ChAT expression showed the opposite pattern. However, after MI diminished expression of ChAT was found most markedly in the SCG-CB area and no clear difference in TH expression was observed (Figure 3A, arrows).

We carried out multiple control experiments to confirm the reliability of the detected co-expression of TH and ChAT in murine SCG neuronal cells. It has previously been demonstrated that a cell bridge, which is present in about 30% of the murine ganglia, connecting the cranial pole of the sympathetic SCG with the parasympathetic nodose ganglion (NG) exists and that the NG is mostly ChAT positive and TH negative (25). To validate our antibody specificity, a double immunofluorescence staining for ChAT and TH was performed on the SCG, the cell bridge and the nodose ganglion (NG). Almost all neuronal cells of the NG were solely positive for ChAT, whereas co-expression of TH and ChAT were observed in the SCG. The cell bridge showed more double positive cells more toward the SCG, and less TH positive cells were observed toward the NG (Supplementary Figure 3A). No obvious differences in ChAT intensity between the SCG and NG could be detected. To validate the presence of ChAT protein in SCG neurons, immunoblot analysis of protein lysates from either whole SCG tissue or isolated nuclei was carried out and showed the presence of a 69KDa protein, in accordance with the presence of ChAT (Supplementary Figures 3B,C). The cell lysate of human epicardial cells, that lack ChAT expression, was used as a negative control (Supplementary Figure 3C). To validate the presence of ChAT mRNA in SCG neurons, HCR-RNA FISH was carried out and shows immunofluorescence representing ChAT mRNA (Supplementary Figure 3D). Lastly, a co-staining of TH and ChAT in a human sympathetic stellate ganglion showed a similar ChAT staining pattern as observed in the murine control ganglion (Supplementary Figure 3E). These findings provide evidence for the presence of ChAT mRNA as well as a 69 kDa ChAT protein, validates the specificity of the ChAT antibody and rules out a cross reaction between ChAT and TH antibodies.

In sympathetic stellate ganglia a phenotypic switch has previously been described, where ChAT expression was downregulated providing a more arrhythmogenic environment (14). Timepoint comparison showed a significant loss or decrease in ChAT intensity of neurons with a significant difference at 24 h after MI, and a similar trend was observed at day 3 and 7 after MI, when compared to control (Figure 3B). Of interest, at day 7 after MI, in 2 out of 5 samples a diminished expression of ChAT in the nuclei was seen, but as this was not visualized in the other 3 out of 5 ganglia, quantification of the overall ChAT positive area within the nucleus throughout the timepoints showed no significant difference (Supplementary Figure 4). As the enlarged neuronal cells displayed a low expression of ChAT, a correlation analysis was performed to examine whether an increase in cell size in the SCG correlated with a decrease of ChAT intensity. This revealed a negative correlation between neuronal cell size and relative ChAT expression, which was significant at 24 h, 3 and 7 days after MI (Figures 3C–F). This correlation disappeared 6 weeks after MI (Figure 3G).

### Brain derived neurotropic factor and nerve growth factor expression is increased in the carotid body and superior cervical ganglia after myocardial infarction

**FIGURE 4:**
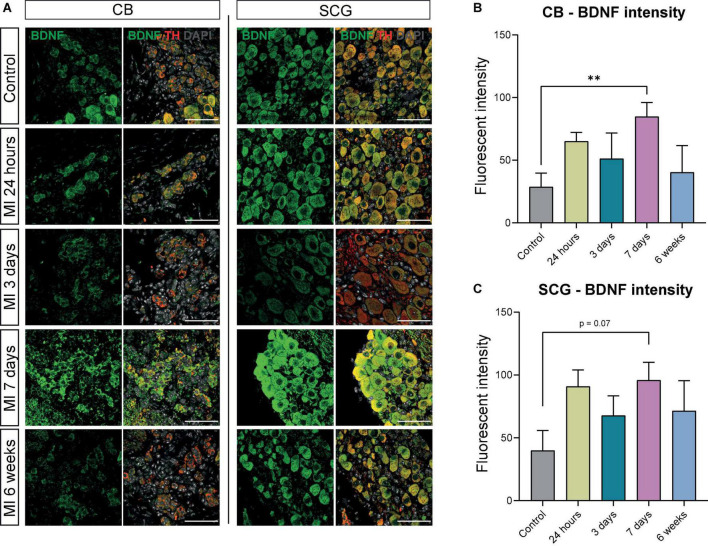
BDNF is increased in CB and SCG neurons after MI. **(A)** Immunofluorescence staining of BDNF (green), TH (red), and DAPI (nuclei, gray) in the CB and SCG of control mice (*n* = 4, 8 SCG) and mice 24 h (*n* = 4, 8 SCG), 3 days (*n* = 2, 4 SCG), 7 days (*n* = 4, 8 SCG), and 6 weeks (*n* = 2, 4 SCG) after MI. Scale bar indicates 50 μm. **(B,C)** Bar graphs display the BDNF intensity of the CB and SCG neurons at different timepoints after MI. Both the CB glomus type I cells and SCG neurons are TH positive cells. ^**^P < 0.01.

The CB type I glomus cells secrete neurotrophic factors during development as well as in response to environmental stimuli (26, 27), therefore we assessed brain derived neurotropic factor (BDNF) and nerve growth factor (NGF) expression in the CB and SCG neurons. Expression of BDNF was present in the CB and SCG neurons at all examined timepoints (Figure 4A). When quantifying the BDNF fluorescent intensity over time, in the CB a significant increase was detected 7 days after MI (Figure 4B). No significant difference could be detected in the SCG neurons in the CB area, although a similar trend was observed (Figure 4C).

**FIGURE 5:**
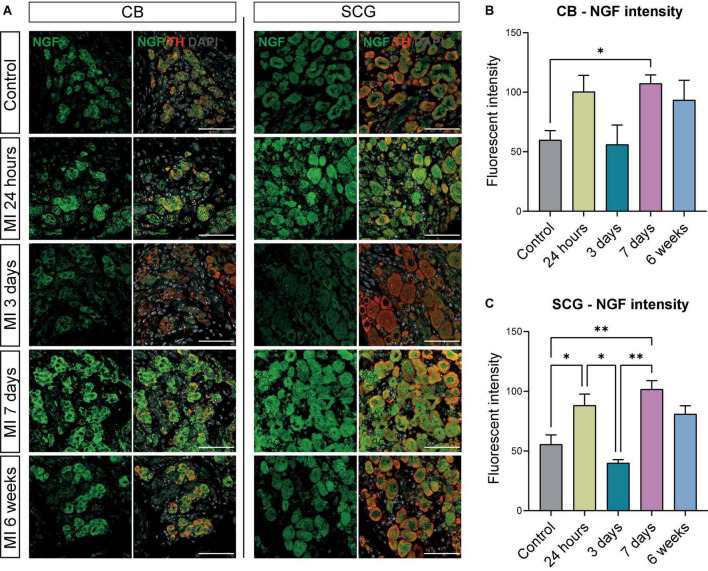
NGF is increased in the CB and neurons in SCG after MI. **(A)** Immunofluorescence staining of NGF (green), TH (red), and DAPI (nuclei, gray) in the CB and SCG neurons of control mice (*n* = 4, 8 SCG) and mice 24 h (*n* = 4, 8 SCG), 3 days (*n* = 2, 4 SCG), 7 days (*n* = 4, 8 SCG), and 6 weeks (*n* = 2, 4 SCG) after MI. Scale bar indicates 50 μm. **(B,C)** Bar graphs display the NGF intensity of the CB and SCG neurons at different timepoints after MI. *P < 0.05, ^**^P < 0.01.

When examining NGF expression at different timepoints, an increase in NGF fluorescent intensity was observed 7 days after MI in the CB and SCG neurons (Figure 5A). These data corresponded with quantification data that showed a significant upregulation of NGF fluorescent intensity in the SCG neurons and the CB at 7 days after MI (Figures 5B,C). In addition, the NGF fluorescent intensity in the SCG neurons showed a significant upregulation 24 h after MI when compared to control, as well as a significant decrease 3 days after MI when compared to 24 h and 7 days after MI (Figure 5C). At 6 weeks after MI, no significant difference as compared to control could be observed anymore.

### High affinity receptors of brain derived neurotropic factor and nerve growth factor in superior cervical ganglia neurons are increased after myocardial infarction

**FIGURE 6:**
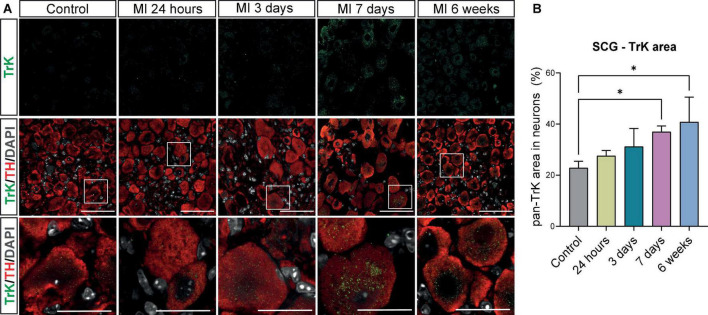
Increase in high affinity receptors for BDNF and NGF in SCG neurons after MI. **(A)** Immunofluorescence staining of pan Trk (green), TH (red), and DAPI (nuclei, gray) in SCG of control (*n* = 4, 8 SCG) mice and mice 24 h (*n* = 4, 8 SCG), 3 days(*n* = 2, 4 SCG), 7 days (*n* = 4, 8 SCG), and 6 weeks (*n* = 2, 4 SCG) after MI. Scale bar indicated 50 μm and 20 μm in the insets. **(B)** Bar graph displays the timepoint comparison of the percentage, the positively stained area out of the total neuronal area, of pan TrK expression in the SCG after MI. *P < 0.05.

The neurotrophic tyrosine receptor Kinase (TrK; e.g., TrKA and TrKB) expression in neurons facilitates the binding of NGF and BDNF and mediates their subsequent impact on neuronal survival and axonal growth (28–30). To study the presence of these receptors in the SCG, immunostaining of the SCG with a pan TrK antibody was carried out and showed a low expression of pan TrK in control SCG neurons (Figure 6A). After MI, pan TrK expression in SCG neurons gradually increased and was significantly upregulated at day 7 after MI. Interestingly, this upregulation persisted at 6 weeks after MI (Figures 6A,B).

### Growth associated protein 43 is upregulated in the superior cervical ganglia after myocardial infarction

**FIGURE 7:**
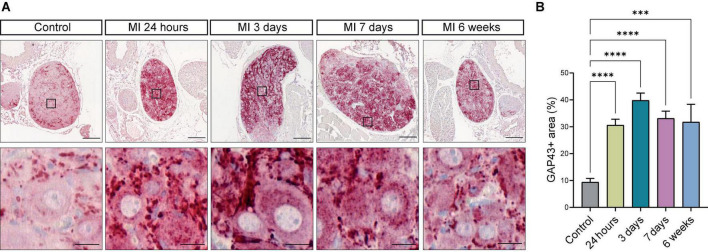
Growth Associated Protein 43 reveals neuronal outgrowth after MI. **(A)** Representative images of a GAP43 immunohistochemistry staining in SCG from control mice (*n* = 4, 8 SCG) and mice 24 h (*n* = 5, 10 SCG), 3 days (*n* = 2, 4 SCG), 7 days (*n* = 5, 10 SCG), and 6 weeks (*n* = 2, 4 SCG) after MI. Scale bar indicates 100 μm and 20 μm in the insets. **(B)** Bar graph displays the timepoint comparison of the percentage, the positively stained area out of the total area, of GAP43 expression in the SCG after MI. ^***^P < 0.001, ^****^P < 0.0001.

As we observed neuronal remodeling concomitant with an increased expression of the neurotrophic factors BDNF, NGF and their receptors, we postulated that this contributes to new axon formation and axonal elongation in the SCG. SCG sections of control mice and mice post-MI were therefore stained for growth associated protein 43 (GAP43), a growth- and plasticity-related protein that is involved in axon elongation and nerve regeneration during early development (31). As shown in Figure 7A, after MI a strong increase in GAP43 expression was observed in neurites and, to a lesser extent, also inside the neuronal cell bodies, while in the control SCG a very low number of neurites expressed GAP43. Timepoint comparison showed a significant upregulation of GAP43 expression at all examined timepoints after MI (Figure 7B). Interestingly, GAP43 was found to be present in the CB as well, Supplementary Figure 5 shows GAP43 staining within the CB at 24 h, 3 days and 6 weeks after MI.

### mRNA levels of brain derived neurotropic factor, nerve growth factor and growth associated protein are elevated 7 days post- myocardial infarction

**FIGURE 8:**
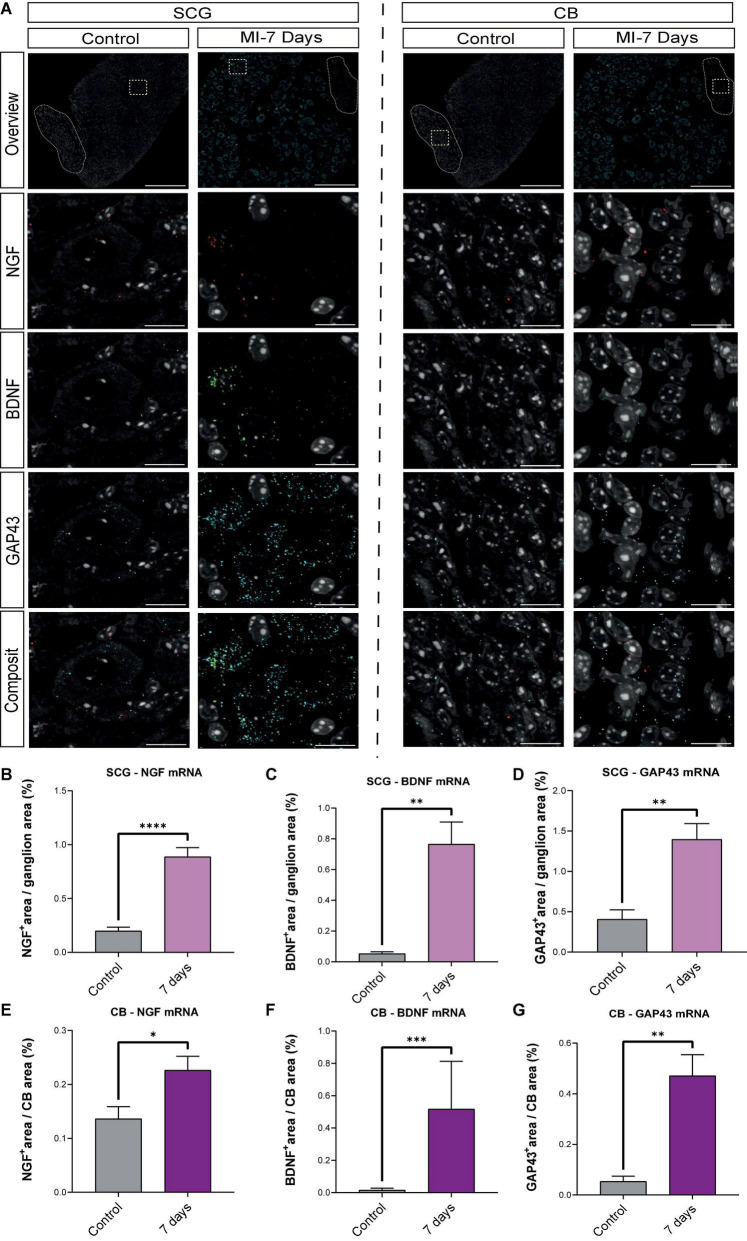
mRNA levels of BDNF, NGF and GAP43 are elevated 7 days post-MI. **(A)** Representative HCR-RNA FISH images of NGF, BDNF and GAP43 in the SCG (left panel) and CB (right panel) in control (*n* = 3, 6 SCG) and 7 days (*n* = 4, 8 SCG) after MI mice. Scale bar indicates 100 μm in the overview panels and 20 μm in the enlarged panels. **(B–D)** Bar graphs display the percentage, the positively stained area out of the total area, of NGF, BDNF and GAP43 in control SCG compared to 7 days after MI. **(E–G)** Bar graphs display the percentage, the positively stained area out of the total area, of NGF, BDNF and GAP43 in control CB compared to 7 days after MI. *P < 0.05, ^**^P < 0.01, ^***^P < 0.001, ^****^P < 0.0001.

To further substantiate our findings, HCR-RNA FISH was performed to evaluate whether the observed changes on a protein level, could also be established at mRNA level. Figure 8A shows representative images of the mRNA content in the SCG neurons (left panel) and CB (right panel). NGF, BDNF and GAP43 are present in both the SCG neurons and CB, where each dot represents a single mRNA molecule. Quantification data in Figures 8B–G show that NGF, BDNF and GAP43 mRNA content are significantly upregulated in both the SCG and CB at 7 days post MI when compared to control.

## Discussion

In the current study, we assessed the neuronal remodeling of murine SCG neurons and the CB at several time points after MI. Key findings are: (i) After MI, neuronal enlargement takes place and the enlargement is amplified in the neurons within the area bordering the CB (referred to as SCG-CB area); (ii) ChAT and TH are co-expressed in SCG neuronal cells, but not in the CB that expresses only TH and not ChAT; (iii) ChAT intensity is significantly downregulated 24 h after MI in SCG neurons; (iv) A significantly negative correlation between neuronal cell size and relative ChAT expression was established in the SCG; (v) Expression of neurotrophic factors BDNF and NGF protein and mRNA was increased in the CB and SCG after MI, concomitant with an increase in their TrK-receptor in the SCG; and (vi) An increased expression of GAP43 protein as well as mRNA, indicative of neuronal remodeling resulting in hyperinnervation after MI.

The role of the autonomic nervous system in post-MI arrhythmogenicity has gained increased attention over the past decades. Whereas vagal innervation is considered cardioprotective, sympathetic overdrive is associated with arrhythmias and sudden cardiac death (32, 33). Remarkably, although nerve tissue is generally notorious for its lack of regeneration capacity in adults, after cardiac damage the intriguing phenomenon of cardiac sympathetic hyperinnervation has been reported in multiple animal species, suggesting a renewed capacity of neuronal outgrowth of sympathetic neurons (34, 35). In line with this, several studies in human, rat, rabbit and pig indicated neuronal and electrical remodeling in the stellate ganglia after MI (9, 13, 14, 36). In addition to the stellate ganglion and upper thoracic ganglia, the cardiac plexus also receives contributions from sympathetic nerves derived from the SCG that participate in cardiac ventricular innervation in both human and mouse (8, 17, 37). However, in contrast to the stellate ganglion, data on the time course of neural remodeling in the SCG – that is bordering the oxygen- and PH- sensing CB – after MI is still limited.

### Expression of choline acetyltransferase in the sympathetic superior cervical ganglia

Sympathetic neuronal cells are classically considered as adrenergic cells, expressing the rate-limiting enzyme TH, that plays a pivotal controlling role in the synthetic pathway of catecholamines (adrenergic neurotransmitters) (38). In contrast, ChAT is the enzyme that catalyzes the synthesis of acetylcholine (cholinergic neurotransmitter in the peripheral nervous system) (39) and is generally considered as a marker for parasympathetic neurons. Remarkably, we observed that almost all neuronal cells co-express TH and ChAT in the murine SCG. The antibodies that were used stained the CB glomus type I cells (TH positive and ChAT negative) and the parasympathetic nodose ganglion (TH negative and ChAT positive), validating the specificity of the TH and ChAT antibodies. This was further supported by the detection of ChAT mRNA and protein, in both the cytoplasm and the nucleus, in the SCG at different timepoints after MI. In addition, neurons co-expressing TH and ChAT were also observed in human sympathetic ganglia in the current study.

These findings contradict with what has been reported in rat and pig sympathetic ganglia, where only few neurons are either bi-phenotypic or ChAT positive (14, 40), and no ChAT expressing neurons were observed in rabbit sympathetic ganglia (13). Of interest, in rat, the presence of an alternative splice variant of common ChAT (cChAT), that lacks exons 6–9, has been shown. This splice variant favors the nerves and neurons within the peripheral nervous system and is therefore called peripheral ChAT (pChat) (41). In the dorsal root ganglia, pChAT has been shown to possess sufficient enzyme activity to supply the neurons with acetylcholine (42). Whether this pChAT has similar functions in the SCG is yet to be determined. These data indicate that phenotype differences in sympathetic neurons among species may exist. Alternatively we hypothesize that, taking into account the different ChAT splice variants, the target of the ChAT antibody may be a determining factor at play. Nevertheless, with regard to the ChAT and TH expression profile, the mouse model seems to resemble human ganglia, thus holding potential as an adequate model to study processes of transdifferentiation (i.e., switch in neuronal phenotype) after cardiac damage, as has been described in animal species as well as in human (43–45).

The SCG does not solely give input to the anterior part of the heart, but has many connections to different glands, vessels and muscles, indicating that it needs to be able to exert multifunctional signals. This is underlined by studies of Matsumoto et al. who detected up to quadruple function, in which these neurons would exert cholinergic, adrenergic, purinergic and non-adrenergic excitatory effects (46). The connection of the SCG to the parasympathetic nodose ganglion has been previously shown and although its function is still unclear, it is considered as a potentially relevant gateway for interaction between sympathetic and parasympathetic neurons (25).

### Neuronal remodeling after acute myocardial infarction

MI timepoint analysis demonstrated neuronal enlargement in the SCG, especially in neurons in the region bordering the CB. Within these neurons a significant decrease in ChAT intensity at 24 h after MI was observed. Remarkably, the level of ChAT showed a negative correlation with neuronal size. We speculate that, as previously shown in heart failure patients, the neuronal enlargement in the SCG are -due to the swelling- harder to excite and thereby contribute to the withdrawal of parasympathetic effects leading to a more pro-arrhythmic environment (47). In addition, we observed a loss of nuclear ChAT in a subpopulation of mice after MI. It has previously been reported that the 69 kDa isoform of ChAT can shift between the cytoplasmic and nuclear compartments. Once located in the nucleus, ChAT can act as a transcriptional activator of high affinity choline transporter 1 (CHT1) which is also involved in the regulation of acetylcholine synthesis in neurons (48). The nuclear localization has also been shown to sustain epigenetic regulations of neuronal structures (49), which might also further mediate the neuronal remodeling observed in the SCG after MI. We speculate that the stress response upon MI induced a loss of nuclear ChAT, which has been observed in 2 out of 5 mice (Supplementary Figure 4), thereby disrupting the balance between autonomic sympathetic and parasympathetic regulation.

### Neurotrophic factors and role of the carotid body

The CB is a neural crest derived structure located at the carotid bifurcation and is the main peripheral chemoreceptor in mammals (26). It can sense and respond to changes in blood flow, O_2_- and CO_2_ levels, PH as well as changes in metabolites such as glucose and lactate (18, 50). Neuron-like glomus cells in the CB express a wide range of growth factors and neurotrophic factors during development (27). In our timepoint analysis, after MI an increase in both protein and mRNA expression of neurotrophic factors (BDNF and NGF) in the CB and SCG was observed. Moreover, an upregulation of the pan TrK (including the high affinity receptors of BDNF and NGF) receptors in the SCG neurons was observed. Surprisingly, the TrK expression in SCG neurons was maintained at high levels at 6 weeks post-MI with a significant difference compared to control (Figure 6B). Results indicate that neuronal remodeling can be influenced by neurotrophic factors via paracrine and/or autocrine effects.

Although neurotropic factors and receptors were upregulated, the question arose whether this could actually contribute to the development of hyperinnervation after MI. GAP43, a growth-associated protein, participates in the developmental regulation of axonal growth and the formation of new synapses, neurite outgrowth, and synaptogenesis after injury (51–53). This might be related to its function in growth cones by stabilizing F-actin, preventing actin polymerization and promoting microtubule-based neurite outgrowth (52, 54, 55). Its transcriptional expression is lowly expressed in mature neurons, but up-regulated in differentiating and regenerating neurons (56). It is thereby a suitable marker to examine the neo-outgrowth during development or after damage. In addition to previous findings of GAP43 expression in sprouting axons in the infarcted heart (57), in the present study we showed a striking upregulation of the GAP43 expression in SCG neurons post-MI starting from as early as 24 h after MI compared to control.

### Strengths and limitations

The strength of this study is that for the first time an elaborate timeline is presented of SCG and CB remodeling post-MI. Key findings have been generated by precise and blinded quantifications with validated antibodies and probes. A limitation is the rather small n-numbers for the different stages, although we strived for a number of at least 8 ganglia for the most relevant stages. As our aim was to study ganglion remodeling after MI at the RNA and protein level, no functional studies were included, and we did not study the paracrine function of neurons or the effects of interventions such as anti-adrenergic therapy or anti-growth factor therapy. Future studies especially aimed at the potential function of ChAT in influencing sympathetic neural activity in the same neurons are warranted.

## Conclusion

In conclusion, neuronal remodeling toward an increased adrenergic phenotype occurs in the SCG and is potentially mediated by the CB. This is substantiated by the marked increase in neuronal cell size of the SCG after MI, especially in the region bordering the CB. A significant decrease in ChAT intensity at 24 h after MI was observed and this coincided with a significantly negative correlation with neuronal size. In addition, upregulation of neurotrophic factors and their high affinity receptors, indicate a paracrine/autocrine neurotrophic effect that is accompanied with an increased upregulation of GAP43 in the SCG. These results suggest an interplay of the SCG and CB after MI, that is likely to contribute to pathological cardiac sympathetic hyperinnervation.

## Future perspectives

In this time-course study, we show that the cholinergic marker ChAT is expressed in sympathetic neurons of the SCG and that expression of ChAT displays a transition in expression after MI. Further studies are required to study the functional implication of ChAT in adrenergic neurons and the mechanisms behind the changes of ChAT caused by MI. Taking into consideration that the CB could influence SCG neuronal remodeling, as was indicated in the present study, the further exploit of the potential interaction between CB and SCG could empower our integrative understanding of cardiac (hyper)innervation after damage.

## Data availability statement

The original contributions presented in the study are included in the article/Supplementary material, further inquiries can be directed to the corresponding author.

## Ethics statement

The animal study was reviewed and approved by the Animal Ethics Committee of the Leiden University, Leiden, Netherlands.

## Author contributions

YG, LR, and MJ designed the experiments. YG, LR, and TG performed the experiments. YG and LR performed data collection and analysis. JG and MJ supervised by data collection and analysis. YG, LR, JG, and MJ wrote the first draft of the manuscript. All authors contributed to the study conception and design and commented on previous versions of the manuscript, and read and approved the final manuscript.

## Footnotes

1
https://www.molecularinstruments.com/
