# Supplementary material for: Acute myocardial infarction induces remodeling of the murine superior cervical ganglia and the carotid body
Source: Front Cardiovasc Med. 2022 Oct 6;9:758265. doi: 10.3389/fcvm.2022.758265 (PMC9582601; doi:10.3389/fcvm.2022.758265)
Supplement: Supplementary file 1 [file Data_Sheet_1.docx]

Supplementary Material

**1. Supplemental material and methods**

**Sirius red staining of MI hearts.**

5 μm-thick paraffin sections of the heart were deparaffinized and stained with Weigert’s Iron Haematoxylin (Merck, HT1079-1SET). Thereafter, the sections were stained with Picrosirius red (0.1% Sirius red (Brunschwig Chemie, 09400) in saturated picric acid (Merck, 197378) for one h. Sections were washed with acidified water (0.5% acetic acid; Merck, 1.00063) and dehydrated prior to mounting with Entellan (Merck, 1.07961). All slides were scanned with the 3D HISTECH Pannoramic 250 scanner. To establish the size of the infarction, the fibrotic area of every 30th section and the total area of the myocardium of every 100th section was measured in ImageJ. These surfaces were multiplied by the difference in thickness to the next measured section and added together to estimate the total volume.

**Immunohistochemistry of β3-tubulin in MI hearts**

For antigen retrieval, 5 μm-thick paraffin sections of the heart were deparaffinized and pre-treated with 0.01 M citric buffer (pH 6.0, 98°C) for 12 minutes. To inhibit endogenous peroxidase, slides were immersed in 0.3% H_2_O_2_ (Sigma-Aldrich 107210) in PBS for 20 minutes. 200 µl of primary antibody, rabbit anti -β3-tubulin (a common nerve marker)(Sigma-Aldrich T2200-200UL; 1:1000), was added to the slides and incubated over-night. On the next day, slides were incubated with goat anti-rabbit-Biotin (Vectorlabs BA-1000; 1:200) together with normal horse serum (Vectorlabs S-2000 ;1:66) for 60 minutes, followed by an incubation with AB-reagent (ABC-HRP kit; PK-6100; Vectorlabs; 1:100) for 45 minutes. To visualise the β3-tubulin staining, slides were immersed in DAB substrate (Sigma-Aldrich D5637) in 400mg/L tris/maleate (pH 7.6) with 4 drops of H_2_O_2_. Finally, the slides were counter stained with haematoxylin (Klinipath VWRK4085-9002) to visualize the nuclei. After dehydration the sections were mounted with Entellan (Merck 107961) mounting medium and all images were captured with the 3D HISTECH Pannoramic 250 scanner.

**Immunofluorescence staining of murine nodose ganglion and human stellate ganglion**

A nodose ganglion was isolated from one mouse 6 weeks after MI and the human stellate ganglion was obtained from a human cadaver. All experiments were performed according to the guidelines of the Leiden University Medical Centre (Leiden, The Netherlands). The 5 μm-thick paraffin sections were deparaffinized, pre-treated with Tris-EDTA buffer (pH 9) at 98°C for 12 min for antigen retrieval, and incubated with anti-tyrosine hydroxylase (TH)(Fisher Scientific PA14679; 1:1000), anti-choline acetyl transferase (ChAT)(Abcam ab181023; 1:1000), and anti-β-tubulin III (β3-tubulin)(Santa Cruz; SC-80005; 1:1000) overnight at 4°C. On the second day, the sections were incubated with the secondary antibodies donkey anti-rabbit Alexa Fluor 488 (Invitrogen A-21206; 1:250), donkey anti-sheep Alexa Fluor 568 (Invitrogen A21099; 1:250) and donkey anti-mouse Alexa Fluor 647 (Invitrogen A31571; 1:250) for 1 h, followed by a 10 min nuclear staining with DAPI (Invitrogen D3571: 1:1000) when indicated. The slides were mounted with ProLong Gold Antifade Mountant (Invitrogen P36930) and images were captured with the Zeiss AxioscanZ1.

**HCR RNA Fluorescent in situ hybridization of ChAT**Hybridization chain reaction RNA fluorescent in situ hybridization (HCR-RNA FISH) was carried out in control and 7 days after MI in SCG sections that contained the CB. The manufacturers protocol was followed and the DNA probes, DNA HCR amplifiers, hybridization buffer and wash buffer were purchased from Molecular Technologies (moleculartechnologies.org)(Choi et al., 2018). Briefly, slides were first heated for 1 h at 60°C to improve adhesion. For RNA retrieval, the slides were deparaffinized and heated in TRIS buffer for 15 minutes at 95°C, followed by a 10 minutes Proteinase K (10 μg/ml) (Promega, V3021) digestion. A humidified chamber was used in all following incubation steps. Probe hybridization with ChAT-B3 (NM_009891.2, LOT PRJ544), II) was performed with 4 pmol/ml probes for 16 h at 37°C. Prior to the hairpin amplification, 6 pmol/ml of the hairpins B3-h1+h2 (fluorophore 488) were snap-cooled by heating to 95°C for 90 seconds and incubated for 30 minutes in the dark at room temperature. Sections were incubated with the hairpin amplifiers for 90 minutes at room temperature. To stain the nuclei, slides were incubated for 10 minutes with DAPI (Invitrogen, D3571; 1:1000) and washed in PBS. Sections were mounted with Prolong Gold Antifade (Invitrogen, P36930) and imaged with a Zeiss Airyscan LSM 900 confocal microscope under the same exposure time and gain settings.

**Western Blotting**
Whole SCG protein was extracted by adding RIPA lysis and extraction buffer (89900; ThermoFisher) supplemented with the protease inhibitor cocktail (87785; ThermoFisher). To extract protein from the nuclear fraction, the SCG were first dissociated by a 30 minute incubation in 2.5% trypsin (25200056; Thermo Fisher Scientific) followed by 30 minute incubation in 5 mg/ml collagenase type II (LS004176; Worthington) at 37°C. After dissociation, the cell suspension was centrifuged at 300rcf for 10 minutes. In order to disrupt the cells, 100 μl of nucleus lysis buffer (10 mM Tris-HCl, 10 mM NaCl, 3 mM MgCl2, and 0.1%Nonidet™ P40 Substitute, 40U/ml RNAse in Nuclease-Free Water) was added onto the cell pellet, mixed well and incubated on ice for 8 minutes. The nuclei suspension was then centrifuged at 300rcf for 5 minutes and washed twice with nucleus wash buffer (1X PBS with 2.0% BSA). RIPA supplemented with Protease inhibitor was added to the nuclei pellet to acquire the nuclear protein content.

For electrophoresis, 20 μg of either whole ganglion or nuclear protein was loaded in 8 % SDS-PAGE gel. Following electrophoretic separation, the proteins were transferred to a polyvinylidene difluoride membrane by wet electroblotting. To block a specific binding, the membrane was incubated with 5% BSA in PBS-0.1% Tween 20 (PBST) for 1 h at RT and incubated with rabbit-anti-ChAT (Abcam ab181023; 1:2000) or sheep-anti-TH (Fisher Scientific PA14679; 1:2000) primary antibodies overnight at 4°C. On the next day, the membrane was incubated for 1 h with HRP-Donkey-anti-Rabbit IgG(H&L) (cell signalling #7074; 1:10,000) or HRP-Donkey-anti-goat IgG(H&L)( Jackson Immuno research 705035003; 1:10,000). The target protein was visualized with a WesternBright kit (Isogen life science). After stripping, the membrane was blocked for 1 h and incubated with rabbit-anti-βtubulin, followed by incubation with HRP-linked antibody as a protein loading reference. Images were captured by C500 western blot imaging system ( Azure biosystems).

**2. Supplemental Figures**

**Supplemental Fig. 1 Fibrosis and hyperinnervation in the heart after MI.**

**A.** The first vertical panel shows representative images of hearts of control (n =4) and 7 days (n=6) after MI stained with Sirius red, which stains fibrosis (red) and healthy myocardium (orange). The second and third vertical panels show a representative section stained with the general nerve marker β3-tubulin, demonstrating hyperinnervation in the area of the myocardial infarction and border zone. Scalebar indicates 1 mm and 50 µm in the insets. **B.** Comparison of the infarction size 1 week and 6 week after MI. ns = not significant.

**Supplemental Fig. 2 Comparison of left and right sided superior cervical ganglia****A and B** comparison of neuronal area between the left and right SCG at 24 h and 7 days post-MI. **C and D** comparison of the ChAT^+^ nuclei/total nuclei area (%) between left and right SCG at 24 h and 7 days post-MI. **E and F** Comparison of the NGF intensity in the SCG neurons between the left and right SCG at 24 h and 7 days post-MI. **G and H** Comparison of the NGF intensity in the CB between the left and right SCG at 24 h and 7 days post-MI.  **I and J** comparison of the BDNF intensity in the SCG neurons between the left and right SCG at 24 h and 7 days post-MI. **K and L** comparison of the BDNF intensity in the CB between the left and right SCG at 24 h and 7 days post-MI. **M and N** comparison of the GAP43 positive area between left and right SCG. ns = not significant.

**Supplemental Fig. 3 Expression of markers of the autonomic nervous system in the murine nodose ganglion, superior cervical ganglion and in the human stellate ganglion.**

**A.** Immunofluorescence staining of TH (red) and ChAT (green) in the murine nodose ganglion (NG), the bridge and superior cervical ganglion (SCG). NG and SCG are indicated by dashed lines. Scale bar indicates 100 μm. **B.** Immunoblot analysis of ChAT or β-tubulin (loading control) in protein lysates of 4 control murine SCGs displaying the expression of ChAT isoforms in SCG. **C.** Immunoblot analysis of ChAT and β-tubulin (loading control) in nuclei protein lysates of 4 SCGs, total cell protein lysates of murine brain (positive control), and total cell protein lysates of human epicardial cells (negative control). Lack of detection of β-tubulin in SCG nuclei lysate confirms that there is no cytoplasmic contamination. **D.** Representative HCR-RNA FISH image of ChAT mRNA (green) and DAPI (nuclei; grey) in SCG neurons. **E.** Immunofluorescence staining of β3-tubulin (grey) TH (red), ChAT (green), and DAPI (nuclei; blue) of a human stellate ganglion, an inset image of merged channels is shown in the right panel. Scale bar indicated 500 μm and 100 μm in the inset.

**Supplemental Fig. 4 Inconsistent expression of ChAT in the nuclei 7 days after MI.**

The expression of ChAT in 5 different mice 7 days after MI. The bar graph displays a timepoint comparison of the expression of ChAT in the neuronal nuclei. ns = not significant

**Supplemental Figure 5. The expression of GAP43 protein in the carotid body.**

Alkaline phosphatase (AP) staining of GAP43 in the CB at 24 h, 3 days and 6 weeks after MI. Scalebar indicated 50 μm .
