# Supplementary material for: Acute myocardial infarction induces remodeling of the murine superior cervical ganglia and the carotid body
Source: Front Cardiovasc Med. 2022 Oct 6;9:758265. doi: 10.3389/fcvm.2022.758265 (PMC9582601; doi:10.3389/fcvm.2022.758265)
Supplement: Supplementary file 2 [file Data_Sheet_2.pdf]

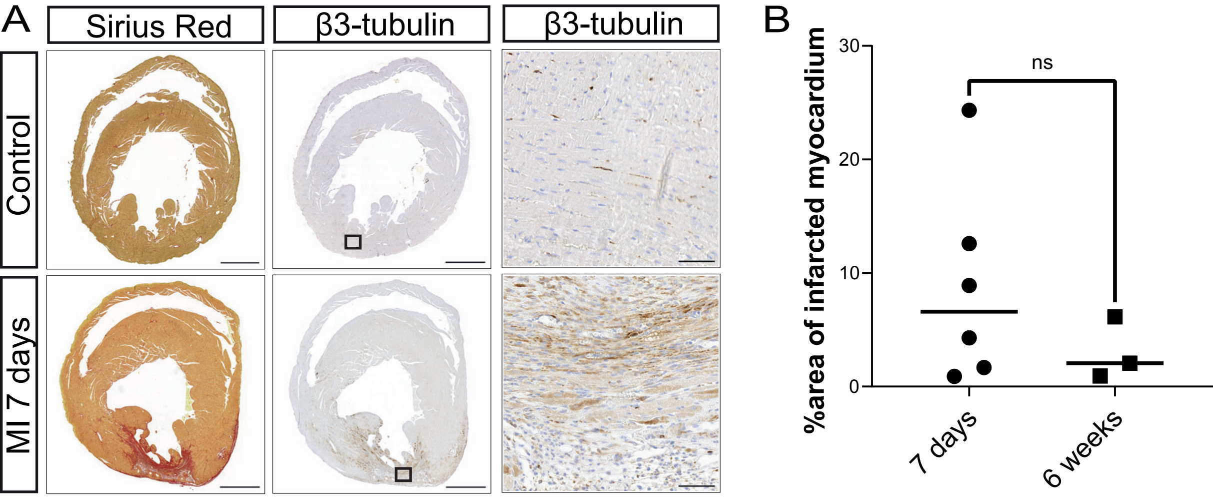
A
Sirius Red
β3-tubulin
β3-tubulin
Control
MI 7 days
B
%area of infarcted myocardium
ns
30
20
10
0
7 days
6 weeks

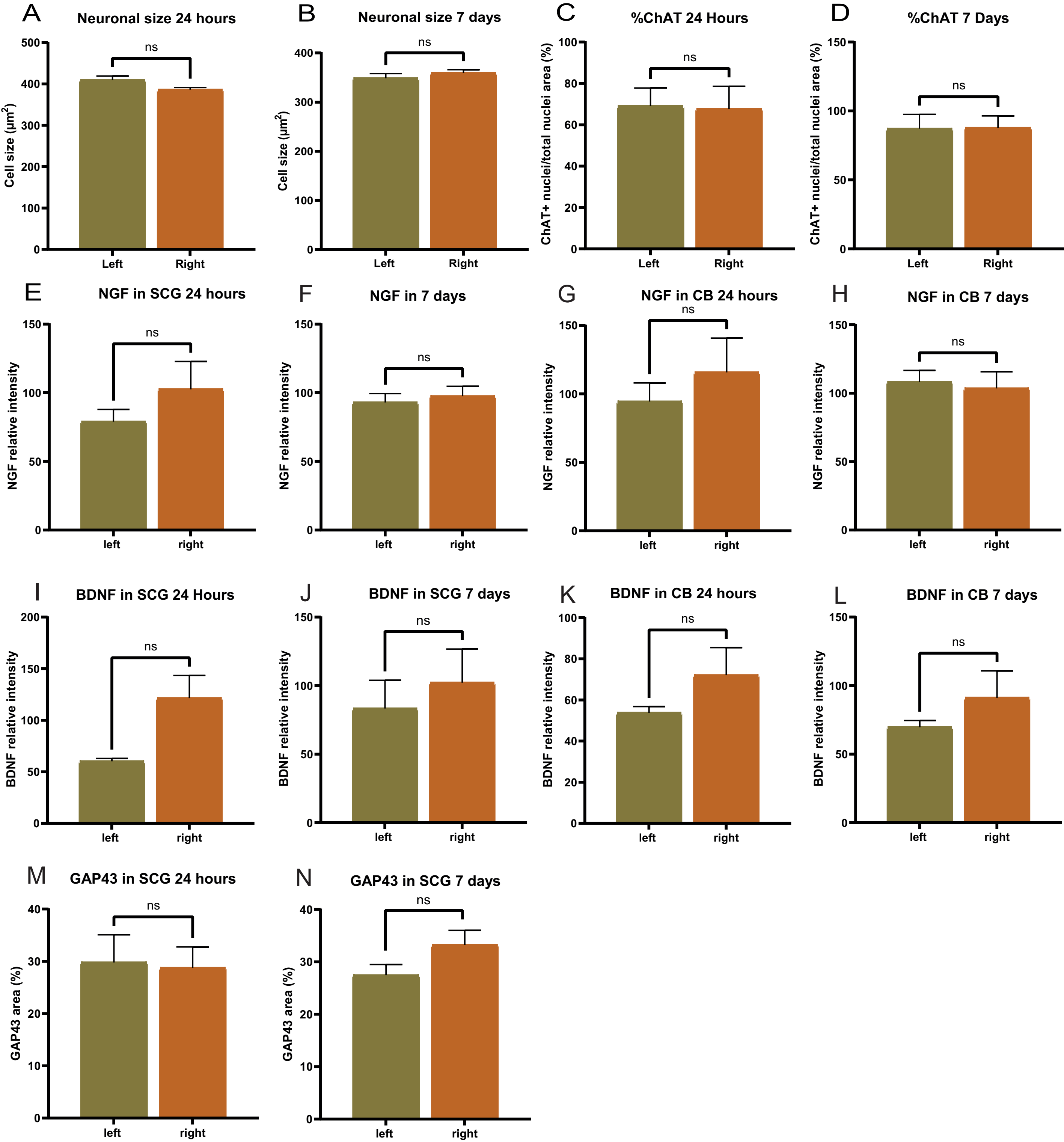
A
Neuronal size 24 hours
Cell size (μm²)
0
100
200
300
400
500
ns
Left
Right
B
Neuronal size 7 days
Cell size (μm²)
0
100
200
300
400
ns
Left
Right
C
%ChAT 24 Hours
ChAT+ nuclei/total nuclei area (%)
0
20
40
60
80
100
ns
Left
Right
D
%ChAT 7 Days
ChAT+ nuclei/total nuclei area (%)
0
50
100
150
ns
Left
Right
E
NGF in SCG 24 hours
NGF relative intensity
0
50
100
150
ns
left
right
F
NGF in 7 days
NGF relative intensity
0
50
100
150
ns
left
right
G
NGF in CB 24 hours
NGF relative intensity
0
50
100
150
ns
left
right
H
NGF in CB 7 days
NGF relative intensity
0
50
100
150
ns
left
right
I
BDNF in SCG 24 Hours
BDNF relative intensity
0
50
100
150
200
ns
left
right
J
BDNF in SCG 7 days
BDNF relative intensity
0
50
100
150
ns
left
right
K
BDNF in CB 24 hours
BDNF relative intensity
0
20
40
60
80
100
ns
left
right
L
BDNF in CB 7 days
BDNF relative intensity
0
50
100
150
ns
left
right
M
GAP43 in SCG 24 hours
GAP43 area (%)
0
10
20
30
40
ns
left
right
N
GAP43 in SCG 7 days
GAP43 area (%)
0
10
20
30
40
ns
left
right

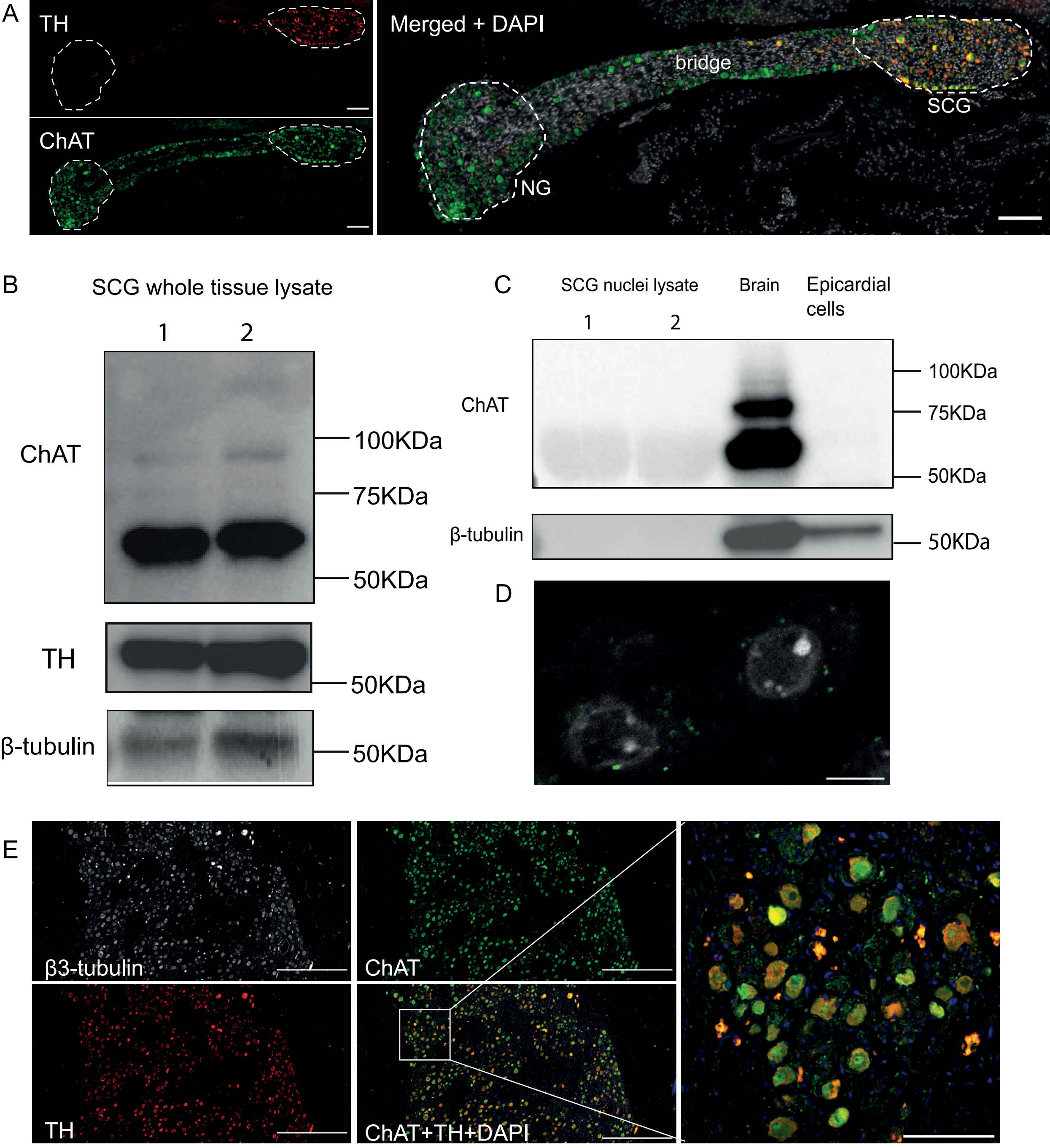
A
TH
ChAT
Merged + DAPI
bridge
SCG
NG
B
SCG whole tissue lysate
1
2
ChAT
100KDa
75KDa
50KDa
TH
50KDa
β-tubulin
50KDa
C
SCG nuclei lysate
Brain
Epicardial cells
1
2
ChAT
100KDa
75KDa
50KDa
β-tubulin
50KDa
D
E
β3-tubulin
ChAT
TH
ChAT+TH+DAPI

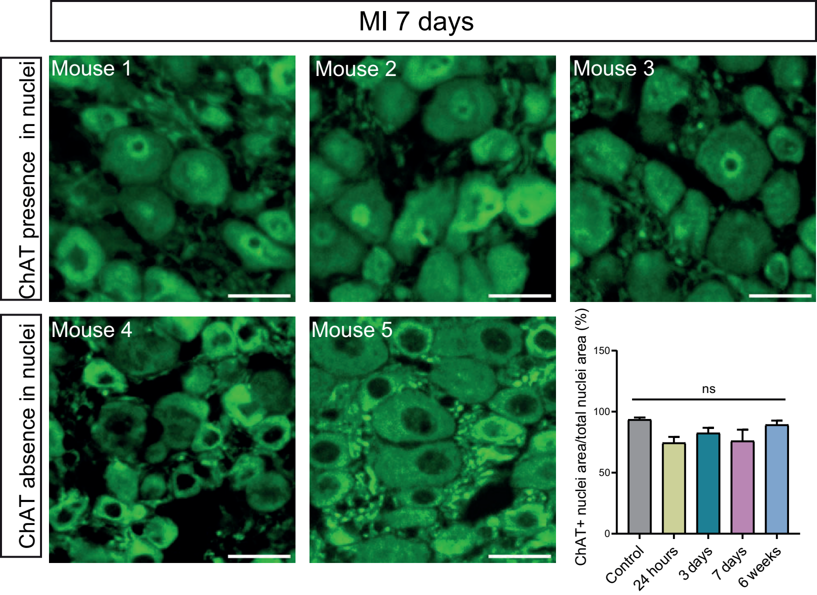

MI 7 days
ChAT presence in nuclei
ChAT absence in nuclei
Mouse 1
Mouse 2
Mouse 3
Mouse 4
Mouse 5
ChAT+ nuclei area/total nuclei area (%)
150
100
50
0
ns
Control
24 hours
3 days
7 days
6 weeks

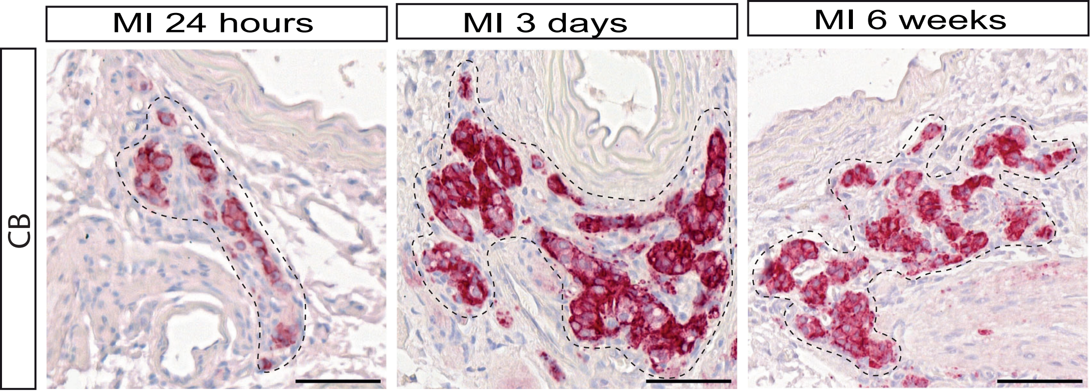
MI 24 hours
MI 3 days
MI 6 weeks
CB
